# RNA-directed DNA methylation requires stepwise binding of silencing factors to long non-coding RNA

**DOI:** 10.1111/tpj.12563

**Published:** 2014-06-23

**Authors:** Gudrun Böhmdorfer, M Jordan Rowley, Jan Kuciński, Yongyou Zhu, Ivan Amies, Andrzej T Wierzbicki

**Affiliations:** Department of Molecular, Cellular and Developmental Biology, University of MichiganAnn Arbor, MI, 48109, USA

**Keywords:** non-coding RNA, DNA methyltransferase, Argonaute, INVOLVED IN DE NOVO2, *Arabidopsis thaliana*

## Abstract

Ribonucleic acid-mediated transcriptional gene silencing (known as RNA-directed DNA methylation, or RdDM, in *Arabidopsis thaliana*) is important for influencing gene expression and the inhibition of transposons by the deposition of repressive chromatin marks such as histone modifications and DNA methylation. A key event in *de novo* methylation of DNA by RdDM is the production of long non-coding RNA (lncRNA) by RNA polymerase V (Pol V). Little is known about the events that connect Pol V transcription to the establishment of repressive chromatin modifications. Using RNA immunoprecipitation, we elucidated the order of events downstream of lncRNA production and discovered interdependency between lncRNA-associated proteins. We found that the effector protein ARGONAUTE4 (AGO4) binds lncRNA independent of the RNA-binding protein INVOLVED IN DE NOVO2 (IDN2). In contrast, IDN2 binds lncRNA in an AGO4-dependent manner. We further found that the *de novo* DNA methyltransferase DOMAINS REARRANGED METHYLTRANSFERASE2 (DRM2) also associates with lncRNA produced by Pol V and that this event depends on AGO4 and IDN2. We propose a model where the silencing proteins AGO4, IDN2 and DRM2 bind to lncRNA in a stepwise manner, resulting in DNA methylation of RdDM target loci.

## Introduction

Eukaryotic genomes contain potentially mobile genomic elements called transposons. As new transposition events can damage genomes, for instance by causing insertion mutations (Belancio *et al*., 2010; Hancks and Kazazian, 2012), transcriptional gene silencing keeps transposons silent. Moreover, this process is important for gene expression, presumably by targeting transposons embedded in the promoters of genes (Zheng *et al*., 2013; Zhong *et al*., 2012; Le Thomas *et al*., 2013; Taliaferro *et al*., 2013). Transcriptional gene silencing may control targets by establishing DNA methylation and other repressive chromatin modifications (Bernstein and Hake, 2006; Grewal and Elgin, 2007; Jiang and Pugh, 2009; Hargreaves and Crabtree, 2011). In *Arabidopsis thaliana*, methylated cytosines are present in three different sequence contexts: symmetrical (CG and CHG, where H stands for any base except G) and asymmetrical (CHH) (Chan *et al*., 2005). After initial methylation of DNA, CG and CHG methylation can be maintained by copying the information from the parental strand after DNA replication. In contrast, CHH methylation needs to be deposited *de novo* after each round of DNA replication in a process called RNA-directed DNA methylation (RdDM). In RdDM, the DNA-dependent RNA polymerase IV (Pol IV) produces a transcript that is converted into double-stranded RNA (dsRNA) by the RNA-dependent RNA Polymerase RDR2 (Xie *et al*., 2004; Herr *et al*., 2005; Kanno *et al*., 2005; Onodera *et al*., 2005; Haag and Pikaard, 2011; Law *et al*., 2011; Wierzbicki, 2012). The resulting dsRNA is processed into 24 nucleotide (nt) long fragments by DICER-LIKE 3 (DCL3) (Xie *et al*., 2004; Kasschau *et al*., 2007) and one strand of this small interfering RNA (siRNA) is bound by the protein ARGONAUTE4 (AGO4) (Qi *et al*., 2006). The siRNA is thought to mediate association of AGO4 via base pairing with a long non-coding RNA (lncRNA), produced by another DNA-dependent RNA Polymerase called Pol V, at the silencing targets (Wierzbicki *et al*., 2008, 2009, 2012; Zheng *et al*., 2013). In addition to AGO4, the lncRNA-binding proteins SPT5L and IDN2 are also important for *de novo* DNA methylation (Ausin *et al*., 2009; He *et al*., 2009; Zheng *et al*., 2010; Zhu *et al*., 2013). Finally, the *de novo* DNA methyltransferase DOMAINS REARRANGED METHYLTRANSFERASE2 (DRM2) is recruited and deposits DNA methyl marks (Cao and Jacobsen, 2002; Naumann *et al*., 2011).

While production of siRNA is generally well understood (Haag and Pikaard, 2011; Wierzbicki, 2012), the order of events downstream of the synthesis of Pol V transcripts is less well studied. So far, AGO4 has been suggested to bind lncRNA in an siRNA-dependent manner, though limited AGO4 stability in *rdr2*, a mutant in siRNA synthesis, weakens this hypothesis (Li *et al*., 2006; Wierzbicki *et al*., 2009). Moreover, IDN2 was shown to act downstream of Pol V and to be important for RdDM, though its exact role remains a mystery. IDN2 was reported to interact with 5′-overhangs of dsRNA *in vitro* (Ausin *et al*., 2009) and, more recently, that it binds Pol V transcripts *in vivo* (Zhu *et al*., 2013). In addition to IDN2, the genome of Arabidopsis encodes IDN2-like (IDNL) 1 and 2 (also called FDM1 and FDM2, respectively) that are both involved in RdDM (Ausin *et al*., 2012; Xie *et al*., 2012a). They were proposed to work with IDN2 in a complex (Ausin *et al*., 2012; Xie *et al*., 2012b) and FDM1 was shown to bind 5′-overhangs of dsRNA *in vitro* similarly to IDN2 as well as unmethylated DNA *in vitro* (Ausin *et al*., 2009; Xie *et al*., 2012a,b). A complex consisting of IDN2 and FDM1 was proposed to simultaneously bind lncRNA and DNA at RdDM targets (Ausin *et al*., 2012; Xie *et al*., 2012b), thereby bringing the Pol V-transcript and the target DNA into closer proximity.

Even though many factors in RdDM have been described (reviewed in Haag and Pikaard, 2011; Wierzbicki, 2012), the order of events downstream of Pol V transcript production and any potential interdependences of lncRNA-binding proteins remain unclear. We discovered that there are two classes of AGO4-bound RdDM targets differing in their dependency on IDN2 or IDN2-like proteins. At loci requiring IDN2, RdDM components bind lncRNA in a certain order downstream of Pol V transcript production. While AGO4 binds to lncRNA independently of IDN2, IDN2 requires AGO4 for its association with Pol V transcripts. These events result in direct or indirect association of DRM2 with lncRNA and methylation of the target DNA.

## Results

### RdDM loci dependent on IDN2 are a distinct set of silencing targets

**Figure 1:**
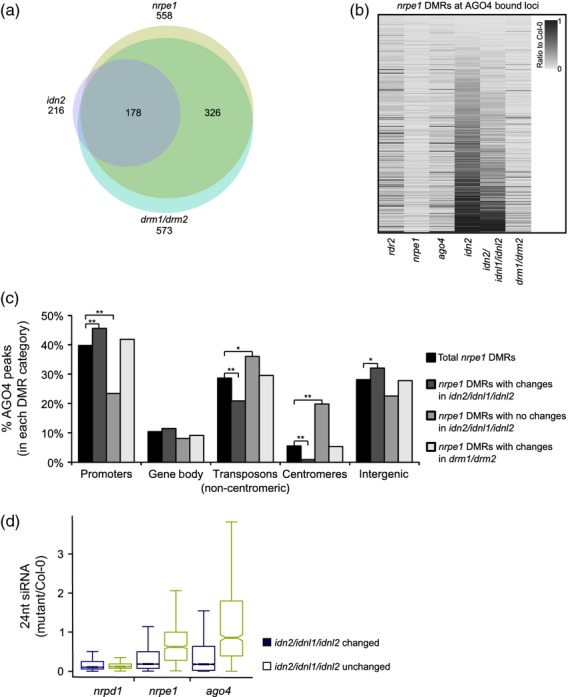
A subset of AGO4-bound RNA-directed DNA methylation (RdDM) targets requires IDN2. (a) Overlaps of AGO4-bound regions of differential CHH methylation (DMRs) between Col-0 and specified mutants. Most *nrpe1* DMRs (beige) also have reduced CHH methylation in *drm1*/*drm2* (blue). These overlap with *idn2* (purple) DMRs. Total DMRs are 558 *nrpe1*, 573 *drm1*/*drm2* or 216 *idn2* out of 820 AGO4-bound loci. (b) Comparison of methylation levels on AGO4-bound *nrpe1* DMRs. Most *nrpe1* DMRs have reduced CHH methylation in *rdr2*, *ago4* and *drm1/drm2*. A subset of *nrpe1* DMRs has reduction in *idn2* or in the *idn2*/*idnl1*/*idnl2* triple mutant, but most have no or intermediate changes. *nrpe1* DMRs were defined as having at least a four-fold reduction in CHH methylation. All values are represented as a ratio to Col-0. (c) Classification of AGO4-bound *nrpe1* DMRs. Any DMRs overlapping promoters of protein-coding genes, gene bodies, transposons, centromeric regions or intergenic regions were counted. Total AGO4-bound *nrpe1* DMRs were plotted as a baseline (black). *nrpe1* DMRs were then divided into regions that have losses in CHH methylation in *idn2*/*idnl1*/*idnl2* (dark grey) and regions that have no change (grey). *nrpe1* DMRs with CHH methylation losses in *drm1*/*drm2* were also plotted (light grey). Bars represent the percentage of each category that overlapped a particular genomic feature. *P <* *0.05, **P <* *0.001 (chi-square test). (d) Production of small interfering RNA (siRNA) on IDN2/IDNL1/IDNL2-dependent regions requires Pol IV, Pol V and AGO4. Total *nrpe1* DMRs were classified as either *idn2*/*idnl1*/*idnl2* changed (blue, meCHH ≤25% of Col-0) or unchanged (green, meCHH ≥75% of Col-0). The 24 nt siRNAs in either *nrpd1*, *nrpe1* or *ago4* overlapping each category were counted and plotted as a ratio to Col-0 with reads per million normalization.

We previously demonstrated that Pol V-mediated binding of AGO4 to chromatin is a general feature of RdDM targets (Zheng *et al*., 2013). To test if CHH DNA methylation on AGO4-bound loci requires other proteins expected to work downstream of Pol V, we analysed published whole-genome bisulfite sequencing datasets (Stroud *et al*., 2013). In agreement with the previously published results (Stroud *et al*., 2013), 90% of the AGO4-bound regions which lost CHH methylation in the *nrpe1* mutant also lost CHH methylation in the *drm1*/*drm2* mutant (Figure1a). This is consistent with DRM1 and DRM2 being the main *de novo* DNA methyltransferases in RdDM (Cao and Jacobsen, 2002; Cao *et al*., 2003). In contrast, only 32% of these loci also lost CHH methylation in the *idn2* mutant (Figure1a), suggesting that IDN2 works only on a subset of RdDM targets.

To test if the IDN2 homologs IDNL1 and IDNL2 compensate for IDN2 in the *idn2* mutant, we compared levels of CHH methylation on AGO4-bound *nrpe1* differentially methylated regions (DMRs). The triple mutant *idn2*/*idnl1*/*idnl2* had lower levels of CHH methylation than the single *idn2* mutant. However, there was still a substantial number of loci with DNA methylation in contrast to the *nrpe1*, *ago4* and *drm1/drm2* mutants (Figure1b). This indicates that only a subset of RdDM targets requires IDN2, IDNL1 or IDNL2 for CHH methylation. Remaining RdDM targets could be silenced independently of IDN2 and its homologs. Alternatively, yet untested IDN2 homologs (Xie *et al*., 2012a) may work at these loci.

To better understand the difference between RdDM targets with IDN2/IDNL1/IDNL2-dependent or -independent CHH methylation, we performed a classification analysis of these regions. The IDN2/IDNL1/IDNL2-dependent loci were more likely to overlap gene promoters, while IDN2/IDNL1/IDNL2-independent targets were more likely to overlap transposons or centromeric regions (Figure1c). Additionally, we compared siRNA production on IDN1/IDNL1/IDNL2-dependent and -independent regions. While only Pol IV was required for siRNA biogenesis at IDN2/IDNL1/IDNL2-independent regions (Figure1d), 24 nt siRNA levels were low in the Pol IV mutant *nrpd1*, the Pol V mutant *nrpe1* and the *ago4* mutant at IDN2/IDNL1/IDNL2-dependent targets, suggesting that siRNA production requires Pol IV, Pol V and AGO4 at these loci (Figure1d). This observation might indicate that IDN2 could be involved in the production of Pol V-dependent siRNAs (Mosher *et al*., 2008; Zheng *et al*., 2010; Lee *et al*., 2012) and that this event requires AGO4.

Together, these results suggest that AGO4-binding sites with IDN2/IDNL1/IDNL2-dependent CHH methylation represent RdDM targets, where all tested silencing factors are involved in agreement with the accepted model of this process (Haag and Pikaard, 2011). Therefore, we selected this category of loci for further analysis of interdependences between proteins involved in RdDM.

### Binding of AGO4 to lncRNA does not require IDN2

To resolve the order of events occurring on Pol V transcripts, we performed RNA immunoprecipitation (RIP) experiments with polyclonal antibodies against AGO4, IDN2 and DRM2. This method allows us to detect RNA bound to the pulled-down proteins after immunoprecipitation. By using appropriate mutant plants, we can determine if an interacting RNA is produced by Pol V and if other proteins are also required for this association. For our assays, we selected four RdDM targets with IDN2/IDNL1/IDNL2-dependent CHH methylation.

**Figure 2:**
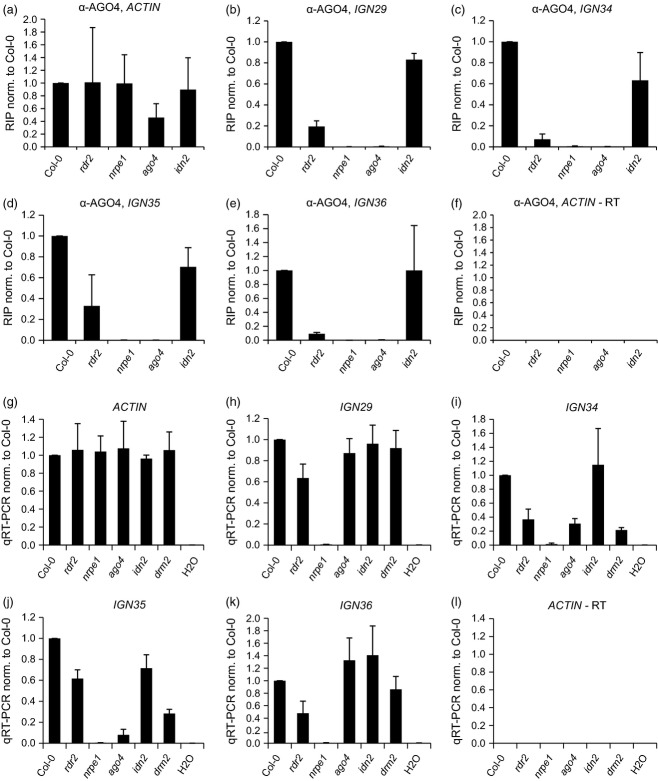
Binding of AGO4 to long non-coding RNA (lncRNA) does not require IDN2. (a)–(f) RNA immunoprecipitation (RIP) experiments using an α-AGO4 antibody and nuclei isolated from wild type and mutant seedlings. Mean values and standard deviations of at least two biological repetitions are shown. Experiments were performed on the following loci: *IGN29* (b), *IGN34* (c), *IGN35* (d), and *IGN36* (e). Pol II-transcribed *ACTIN2* served as a control for equal total RNA levels (a) and for potential contamination with genomic DNA (f). (g–l) Quantitative reverse-transcriptase polymerase chain reaction (qRT-PCR) with total RNA isolated from seedlings. Mean values and standard deviations of three biological repetitions are shown. Four Pol V-transcribed loci with reduced DNA methylation in *idn2* were analysed: *IGN29* (h), *IGN34* (i), *IGN35* (j), and *IGN36* (k). Pol II-transcribed *ACTIN2* served as a control for equal total RNA levels (g) and potential contamination with genomic DNA (l).

To determine if the binding of AGO4 to lncRNA depends on IDN2, we performed RIP experiments with an α-AGO4 antibody using seedlings of Col-0 wild type, the *idn2* mutant as well as *nrpe1*, *ago4* and *rdr2* mutants. Non-specifically immunoprecipitated *ACTIN2* RNA was used to monitor for comparable levels of total RNA in the samples (Figure2a). Quantitative (q)RT-PCR reactions without reverse transcriptase allowed detection of potential contamination with genomic DNA (Figure2f). RNA immunoprecipitation using Col-0 wild type pulled down RNA from all four of the tested loci, while we observed a loss of this signal in the *ago4* mutant. This result indicates that AGO4 associates with RNA at these loci (Figure2b–e). The signal was not detectable in the *nrpe1* mutant either (Figure2b–e), indicating that the transcripts that AGO4 interacts with are produced by Pol V. Recovery of RNA was strongly reduced in the *rdr2* mutant, which suggests that siRNA is required for the association of AGO4 with Pol V transcripts. This result is consistent with data obtained at other loci (Wierzbicki *et al*., 2009); however, reduced stability of AGO4 in the *rdr2* mutant (Li *et al*., 2006; Wierzbicki *et al*., 2009) makes this result difficult to interpret. In contrast, levels of immunoprecipitated Pol V transcripts were not significantly changed in *idn2* compared with the wild type (Figure2b–e), indicating that IDN2 is not important for the interaction of AGO4 with lncRNA.

To test if altered Pol V transcript levels in any of the tested mutants are affecting the results of the RIP assay, we performed qRT-PCR on total RNA. Loss of qRT-PCR signal in *nrpe1* demonstrated that all four loci are indeed transcribed by Pol V (Figure2h–k). Levels of Pol V transcripts were not significantly changed in *idn2* at any of the tested loci, while they were slightly reduced in *rdr2*. For *ago4* and *drm2* mutants, the amounts of Pol V transcripts were not substantially altered at *IGN29* nor at *IGN36* (Figure2h,k), while they were partially reduced at two other loci (Figure2i,j). These results indicate that despite some limited locus-specific variability, differences in the accumulation of Pol V transcripts between tested mutants do not account for the effects observed in the RIP experiments.

In summary, the association of AGO4 with Pol V transcripts at the tested loci does not require IDN2. This suggests that IDN2 works downstream of or in parallel to the binding of AGO4 to lncRNA.

### Binding of IDN2 to lncRNA requires siRNA and AGO4

**Figure 3:**
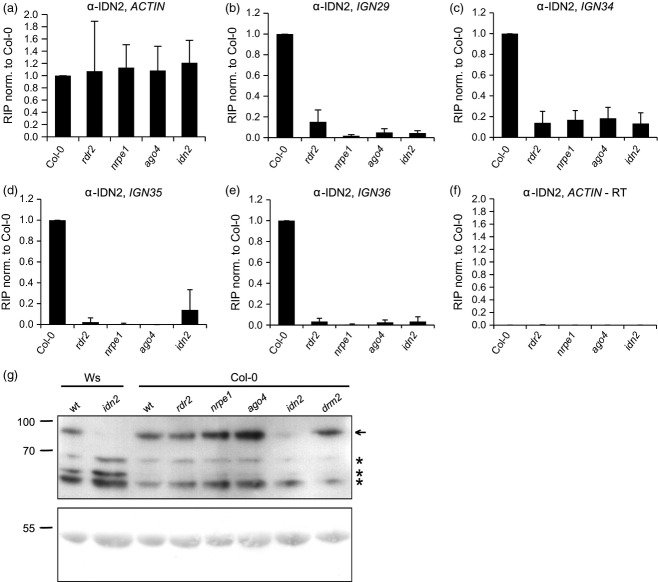
Binding of long non-coding RNA (lncRNA) by IDN2 depends on small interfering RNA (siRNA) and AGO4. (a–f) RNA immunoprecipitation (RIP) experiments with an α-IDN2 antibody and nuclei isolated from wild type and RNA-directed DNA methylation (RdDM) mutant seedlings. Mean values and standard deviations of at least three biological replicates are shown. Experiments were performed on the following loci: *IGN29* (b), *IGN34* (c), *IGN35* (d), and *IGN36* (e). Pol II-transcribed *ACTIN2* served as a control for equal total RNA levels (a) and for potential contamination with genomic DNA (f). (g) Western blot with an α-IDN2 antibody and proteins isolated from wild type (wt) and RdDM mutant seedlings. Ponceau staining of the membrane shows comparable loading of protein extracts. The experiment was performed twice and one representative biological replicate is depicted.

Even though binding of lncRNA by AGO4 does not seem to require IDN2, the association of IDN2 with Pol V transcripts might need AGO4. Alternatively, both proteins could bind lncRNA independently of each other. To distinguish between these two scenarios and to determine if IDN2 actually binds Pol V transcripts at studied loci, we performed RIP experiments with an α-IDN2 antibody in the Col-0 wild type, *rdr2*, *nrpe1*, *ago4* and *idn2*. While immunoprecipitated RNA was detectable in Col-0, qRT-PCR signals were strongly reduced in the *idn2* mutant (Figure3b–e), indicating that IDN2 associates with the tested RNAs. The signal was also lost in the *nrpe1* mutant (Figure3b–e), which shows that IDN2 binds Pol V transcripts. No RNA above the *idn2* background level was recovered from *rdr2* or *ago4* mutants (Figure3b–e). This result indicates that binding of IDN2 to Pol V transcripts requires AGO4 and potentially siRNAs, although the lower RIP signal in *rdr2* may be explained by reduced protein levels of AGO4 in *rdr2* as well.

To test if levels of the IDN2 protein are comparable between studied mutants, we performed Western blots with the α-IDN2 antibody. Specific signal was not detectable in the knock-out *idn2-2* mutant (Ws background) and strongly reduced in the *idn2-1* mutant (Col-0 background), confirming antibody specificity. The IDN2 protein levels were not significantly changed in any of the other RdDM mutants tested when compared with the wild type (Figure3g), indicating that results of RIP are not caused by differences in IDN2 protein accumulation. Therefore, we conclude that binding of IDN2 to lncRNA is dependent on siRNA and AGO4. This suggests that IDN2 works downstream of AGO4.

### The DRM2 protein associates with lncRNA produced by Pol V

**Figure 4:**
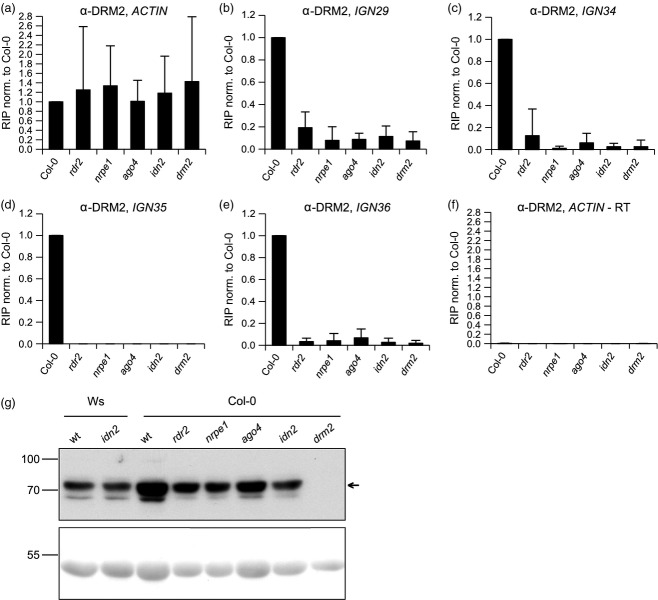
Association of DRM2 with Pol V transcripts requires small interfering RNA (siRNA), AGO4 and IDN2. (a–f) RNA immunoprecipitation (RIP) experiments with an α-DRM2 antibody and nuclei isolated from wild type and RNA-directed DNA methylation (RdDM) mutant seedlings. Mean values and standard deviations of at least two biological replicates are shown. Experiments were performed on the following loci: *IGN29* (b), *IGN34* (c), *IGN35* (d), and *IGN36* (e). Pol II-transcribed *ACTIN2* served as a control for equal total RNA levels (a) and for potential contamination with genomic DNA (f). (g) Western blot with an α-DRM2 antibody and proteins isolated from wild type (wt) and RdDM mutant seedlings. Ponceau staining of the membrane shows comparable loading of protein extracts. The experiment was performed twice and one representative biological replicate is depicted.

Experiments described above as well as previously published work (He *et al*., 2009; Wierzbicki *et al*., 2009; Rowley *et al*., 2011; Zhu *et al*., 2013) have demonstrated that three proteins (AGO4, SPT5L and IDN2) found to work downstream of Pol V actually associate with Pol V transcripts. Therefore, we hypothesized that DRM2 may also bind lncRNA produced by Pol V. To test this hypothesis, we performed RIP experiments with an α-DRM2 antibody. We were able to amplify RNA from the Col-0 wild type at all four tested loci, while the signal was undetectable or was strongly reduced in the *drm2* mutant (Figure4b–e), indicating that DRM2 associates with RNA at these loci. The signal was also undetectable or strongly reduced in the *nrpe1* mutant (Figure4b–e). Therefore, we concluded that that DRM2 associates specifically with Pol V transcripts at these loci.

### The DRM2 protein requires AGO4 and IDN2 to bind to lncRNA

The association of DRM2 with lncRNA may be independent of the two other lncRNA-binding proteins IDN2 and AGO4. Alternatively, DRM2 might require prior association of these two proteins as well as the presence of siRNA. To test these possibilities, we included the *rdr2*, *ago4* and *idn2* mutants in our analysis. The α-DRM2 RIP experiments showed a similar loss of qRT-PCR signal in the *rdr2*, *ago4* and *idn2* mutants compared with *drm2* and *nrpe1* (Figure4b–e), suggesting that binding of DRM2 to Pol V transcripts requires AGO4, IDN2 and siRNA. To test if the observed effects were caused by reduced stability of DRM2 in the studied mutants, we performed a Western blot with the α-DRM2 antibody on total protein extracts. A specific signal was not detectable in the *drm2* mutant, confirming antibody specificity. In the other tested mutants, levels of DRM2 protein were comparable to those in the Col-0 wild type (Figure4g), indicating that the results from the RIP assays are not caused by altered protein levels in RdDM mutants. Together, these results suggest that the association of DRM2 with Pol V transcripts occurs downstream of AGO4 and IDN2.

### Association of AGO4, IDN2 and DRM2 with Pol V transcripts is important for CHH DNA methylation

**Figure 5:**
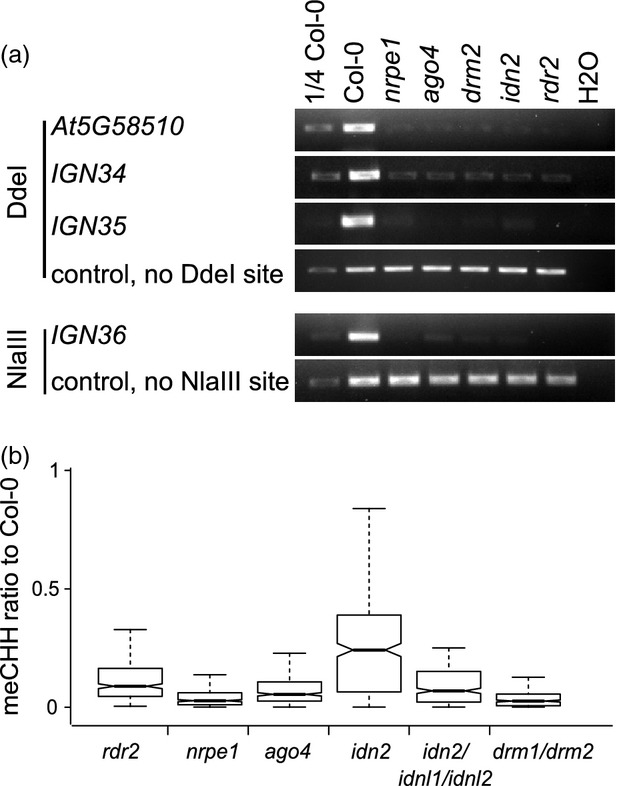
Pol V transcripts, small interfering RNA (siRNA), AGO4, IDN2 and DRM2 are necessary for CHH methylation at IDN2-dependent loci. (a) Chop-PCR with genomic DNA isolated from wild type and RNA-directed DNA methylation (RdDM) mutant seedlings. The DNA was digested with methylation-sensitive restriction enzymes and amplified by PCR. Three Pol V-transcribed loci with reduced DNA methylation levels in *idn2* were analysed in addition to a control locus (*At5G58510*) that has been previously shown to have Pol V- and AGO4-dependent CHH methylation (Zheng *et al*., 2013). Regions lacking a restriction site were used as a loading control. The experiment was performed three times and one representative biological replicate is shown. (b) Methylation status of AGO4-bound *nrpe1* differentially methylated regions (DMRs) as classified by the *idn2*/*idnl1*/*idnl2* triple mutant. Those DMRs with a dependency on *idn2*/*idnl1*/*idnl2* were selected and methylation levels in *rdr2*, *ago4*, *idn2* and *drm1/drm2* relative to the wild type were plotted. Dependency was defined as at least a four-fold reduction in CHH methylation compared with Col-0.

Our RIP experiments showed that AGO4, IDN2 and DRM2 bind lncRNA at AGO4-bound DMRs. If this event is important for CHH methylation, DNA methylation levels should be reduced in all the mutants at these loci. To test this hypothesis, we performed Chop-PCR experiments in all the mutants as well as in the wild type. In a Chop-PCR assay, genomic DNA is digested with methylation-sensitive restriction enzymes and subsequently amplified by PCR. If DNA methylation is present, the enzyme cannot cut and a PCR product can be amplified. If DNA methylation is lost, then the enzyme digests the DNA and no PCR product can be observed. At all tested loci DNA methylation levels were reduced to a similar extent in the *rdr2*, *nrpe1*, *ago4*, *idn2* and *drm2* mutants (Figure5a), indicating that siRNAs produced by RDR2, Pol V transcripts, AGO4, IDN2 as well as DRM2 are all required for CHH methylation at these regions.

We also tested the relationship between DNA methylation levels in tested mutants throughout the genome. To do so, we analysed published datasets (Stroud *et al*., 2013) and compared all genomic regions where DNA methylation was reduced in the *idn2*/*idnl1*/*idnl2* triple mutant. These regions had consistently strong reductions of CHH methylation levels in *rdr2*, *nrpe1*, *ago4* and *drm1/drm2* mutants (Figure5b). Together, these results suggest that all of the RdDM components included in the RIP experiments are important for DNA methylation at the studied category of loci. This is consistent with our results suggesting that AGO4, IDN2 and DRM2 are working together in a stepwise fashion to establish CHH DNA methylation.

## Discussion

We have previously shown that association of AGO4 with Pol V-produced lncRNA is necessary for binding of AGO4 to chromatin and subsequent methylation of DNA throughout the genome (Wierzbicki *et al*., 2009; Zheng *et al*., 2013). To test if other RdDM components acting downstream of Pol V (i.e. IDN2 and DRM2) work in a more locus-specific way, we analysed published DNA methylation datasets (Stroud *et al*., 2013), focusing on AGO4-bound genomic regions. While DRM1/DRM2 (as expected) is required for CHH methylation at these loci, IDN2 and IDN2-related proteins are not always required for RdDM. In fact, there are two distinct categories of RdDM targets differing not only in their dependency on IDN2/IDNL1/IDNL2 but also in their location (Figure1c) and in the involvement of other RdDM factors in siRNA biogenesis (Figure1d). In particular, IDN2-dependent targets are enriched in promoters and intergenic regions while they are depleted in transposons and centromeres. It is possible that other IDN2-related proteins (Xie *et al*., 2012a; Butt *et al*., 2014) with a different preference for target loci might compensate for IDN2 at these loci. Alternatively, the function of IDN2 or IDN2-related proteins might not be required at these targets. For instance, the repetitive nature of transposons and centromeric sequences might already give rise to dsRNA that could be further processed by a dicer protein. In contrast, at non-repetitive regions such as promoters and intergenic sequences, IDN2 might be required for second-strand synthesis of non-coding RNA produced by Pol V, as indicated by our analysis of 24 nt siRNA on IDN2-dependent and independent regions (Figure1d).

**Figure 6:**
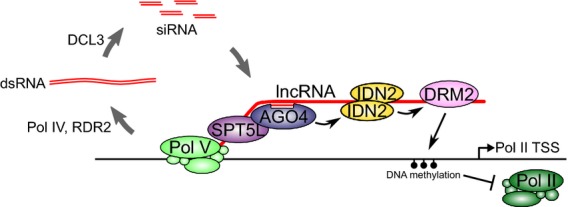
Model of stepwise association of silencing proteins with long non-coding RNA (lncRNA) produced by Pol V to mediate CHH methylation. At IDN2/IDNL1/IDNL2-dependent loci, AGO4 interacts with lncRNA produced by Pol V in a manner at least partially dependent on small interfering RNA (siRNA). In turn, IDN2 binds to lncRNA possibly recognizing a 5′-overhang of a double-stranded RNA consisting of siRNA and lncRNA. Finally, DRM2 directly or indirectly associates with the Pol V transcript in a way dependent on IDN2 and AGO4, and the DNA at the locus is methylated.

As we wanted to understand the order of events downstream of Pol V production, we focused our study on IDN2/IDNL1/IDNL2-dependent loci and asked if lncRNA produced by Pol V at representative loci is bound by AGO4, IDN2 and DRM2. We also determined if these proteins bind to lncRNA in a particular order. We first tested if IDN2 is required for recruiting AGO4 to RdDM targets using RIP with an α-AGO4 antibody. While the experiments revealed that IDN2 is not essential for binding of lncRNA by AGO4, we confirmed that siRNA produced by RDR2 seems to be important for this event, as previously suggested (Wierzbicki *et al*., 2009; Zheng *et al*., 2013). Therefore, AGO4 binds to lncRNA independently of IDN2 (Figure6).

As IDN2 is not required for the association of AGO4 with lncRNA, it might work either downstream or in parallel to AGO4. To distinguish between these possibilities, we performed α-IDN2 RIP experiments. The results suggest that binding of IDN2 to Pol V transcripts requires AGO4 and siRNA (Figure6). As IDN2 protein levels are not changed in any of the mutants tested besides *idn2*, this result could be explained by siRNA being primarily important for the association of AGO4 with lncRNA via base pairing. In turn, AGO4 could interact with IDN2, thereby recruiting it to Pol V transcripts. So far, no direct interaction has been observed between AGO4 and IDN2 in purified protein complexes (Ausin *et al*., 2012). However, an interaction between FDM1 and AGO4 mediated by RNA has been reported (Xie *et al*., 2012a), suggesting simultaneous association of AGO4 and an IDN2 complex containing FDM1 with RNA. As IDN2 was reported to bind 5′-overhangs of dsRNA (Ausin *et al*., 2009), AGO4 might deposit the siRNA on the lncRNA. Then, IDN2 could bind to the resulting dsRNA now containing a 5′-overhang, as was proposed previously (Ausin *et al*., 2012).

As IDN2 and AGO4, two proteins working downstream of Pol V production, were shown to bind lncRNA, we wanted to test if DRM2 also associates with Pol V transcripts. Our RIP experiments demonstrated that DRM2 binds Pol V transcripts in an AGO4- and IDN2-dependent manner. We therefore propose that AGO4 binds a Pol V transcript in an siRNA-guided manner followed by association of IDN2 with subsequent recruitment of DRM2 (Figure6).

Our experiments demonstrating the binding of DRM2 to lncRNA involved formaldehyde crosslinking, which may induce covalent bond formation not only between proteins and nucleic acids but also between proteins and proteins. Therefore, we cannot distinguish between DRM2 directly binding to Pol V transcripts or requiring a lncRNA-binding protein that mediates an indirect association of DRM2 to lncRNA. Future studies will be necessary to address this question as we were unsuccessful in our preliminary attempts to replace formaldehyde with UV crosslinking, which specifically fixes protein–nucleic acid interactions. Similarly, tests for potential protein–protein interactions between IDN2 and DRM2 by yeast-two-hybrid and transient expression in tobacco were inconclusive. Moreover, no direct protein–protein interaction between FDM1 and DRM2 has been reported (Xie *et al*., 2012b). So far only an indirect association between AGO4 and DRM2 mediated by RDM1 has been proposed (Gao *et al*., 2010). However, Pol V transcripts are reduced in the *rdm1* mutant (Law *et al*., 2010), which makes *in vivo* interactions of this protein with Pol V transcripts hard to study.

It is possible that DRM2 interacts directly with Pol V transcripts, which would be consistent with reports from other organisms (Mohammad *et al*., 2010; Schmitz *et al*., 2010; Holz-Schietinger and Reich, 2012; Di Ruscio *et al*., 2013). In murine cells, Dnmt3b was suggested to specifically recognize DNA:RNA triplexes (Schmitz *et al*., 2010), while human DNMT3A and DNMT1a were reported to interact directly with a specific RNA (Holz-Schietinger and Reich, 2012; Di Ruscio *et al*., 2013). This binding event can depend on specific elements of a RNA, as murine Dnmt1 was observed to bind a certain region of a particular lncRNA in order to be recruited to DNA methylation targets (Mohammad *et al*., 2010). Similarly, DRM2 might recognize and bind distinct regions or structural features of lncRNA. It is therefore feasible that AGO4 binds to Pol V transcripts and deposits siRNAs, which in turn establish 5′-overhangs that are recognized by IDN2. Binding of IDN2 could introduce a conformational shift in the RNA that could allow association of DRM2. Alternatively, this interaction could require IDN2 to somehow stabilize Pol V transcripts, similarly to SGS3 stabilizing precursors for siRNA (Yoshikawa *et al*., 2005; Fukunaga and Doudna, 2009). However, we did not observe any major changes in Pol V transcript levels in the *idn2* mutant (Figure2h–k), suggesting that IDN2 probably does not affect the stability of lncRNA. As a third possibility, IDN2 could be necessary to prime second-strand synthesis of Pol V transcripts by an RNA-dependent RNA polymerase. This would be consistent with our analysis of Pol V-dependent siRNA, the partial reduction of siRNA levels on some RdDM targets in the *idn2* mutant (Zheng *et al*., 2010) and the observation that FDM1/FDM2 is important for the synthesis of Pol V-dependent siRNAs (Xie *et al*., 2012a). Hence, DRM2 could hypothetically recognize a dsRNA primed by an IDN2–FDM1 complex. Future experiments elucidating what happens on Pol V transcripts could provide further insights into these open questions.

## Experimental procedures

### Plant material

*nrpe1* (*nrpd1b-11*) and *ago4* [*ago4-1* (Zilberman *et al*., 2003) introgressed into the Col-0 background] have been described previously (Onodera *et al*., 2005; Wierzbicki *et al*., 2009). *idn2-1* (Ausin *et al*., 2009) was kindly provided by Steven Jacobsen (UCLA/MCDB, Los Angeles, CA, USA). *idn2-2* in the Ws background (FLAG_550B05) was obtained from INRA. *drm2-2* (SAIL_70_E12) was described previously (Wierzbicki *et al*., 2008).

### Bioinformatics analysis of DMRs

The genome-wide bisulfite sequencing data used were previously reported by Stroud *et al*. (2013) (GEO accession number GSE39901). Differentially methylated regions (DMR) were defined as having <25% methylation in the mutant versus wild type on AGO4 peaks (Zheng *et al*., 2013). The weighted Venn diagram was created using the Venneuler package in R. A list of AGO4 peaks with DNA methylation data is provided in Table S1 in Supporting Information.

To create a heatmap, AGO4 peaks with a CHH methylation signal present in Col-0 and *nrpe1* to Col-0 CHH methylation ratio of <0.25 were kept. Relative methylation signals on these peaks were plotted in R as a mutant/Col-0 ratio with Col-0 as the maximum signal.

AGO4 peaks with a *nrpe1* to Col-0 CHH methylation ratio of <0.25 were further divided into *idn2/idnl1/idnl2* changed (<25% of Col-0) and unchanged (>75% of Col-0) DMR categories. These categories were intersected with TAIR10 annotated genomic features, counted and plotted.

Small RNA with 24 nt that overlapped these *nrpe1* DMR categories (*idn2*/*idnl1*/*idnl2* changed and unchanged) were counted and plotted as boxplots with the ratio of siRNA of each mutant to Col-0. The mutants examined for siRNA were *nrpd1*, *nrpe1* (SRA accession number SRA054962; Wierzbicki *et al*., 2012) and *ago4* (GEO accession number GSE16545; Havecker *et al*., 2010).

### Quantitative RT-PCR and RIPs

The RNA immunoprecipitation experiments were performed with 3 g of approximately 3-week-old seedlings fixed with 0.5% formaldehyde as described (Rowley *et al*., 2013). Values were normalized to the wild type. Experiments were performed in at least two biological replicates.

For qRT-PCR, total RNA was isolated from approximately 3-week-old seedlings using the SV Total RNA Isolation kit from Promega (http://www.promega.com/) according to manufacturer's recommendations. In addition to the on-column DNase I digestion, the RNA was digested with Turbo DNase as described (Rowley *et al*., 2013). Three micrograms of total RNA was used for cDNA synthesis with random primers (Invitrogen, http://www.invitrogen.com/). The means and standard deviations were obtained from three biological replicates. The number of test loci was determined by the amount of RNA recovered in RIP and the requirement to perform all assays in multiple biological repeats. The following primer pairs were used: *IGN29* JA227 CGTTTGTTTATGTAGGGCGAAAG and JA228 TAAAACTTTTCCCGCCAACCA (Zhu *et al*., 2013), *IGN34* GB396 ATGAATAACAAATTTGGAGTCGTC and GB397 CCCTTTCATCGACACTGACA, *IGN35* GB588 GACGGACCAAACGATTTCAT and GB589 TTCCTCTTTGAGCTTGACCA, *IGN36* GB646 CAGTTTTGGGTGCGGTTTAT and GB647 GACAAAAATTGCTTTAGACCATGA.

### Antibody production

A DRM2 fragment (amino acids 29–423, amplified with the following primers: DRM2F1 CACCATGCAGTGTAGGGTCGAAAATCTAGCT and DRM2R1 CTAATGCTTAGGCGGTTCTGGTTCTTC) was used for production of a polyclonal antibody in rabbits. The antibody was purified as described in Zhu *et al*. (2013). α-IDN2 and α-AGO4 antibodies were described previously (Wierzbicki *et al*., 2009; Zhu *et al*., 2013).

### Western blots

Total protein extracts were isolated from 100 mg of approximately 3-week-old Arabidopsis seedlings and separated on 12% polyacrylamide gels. Western blots were performed using α-IDN2 (1:333) and α-DRM2 (1:500) antibodies. Donkey anti-rabbit secondary antibody (1:2000) conjugated with horseradish peroxidase was used (Santa Cruz Biotechnology, http://www.scbt.com/).

### Chop-PCR

Genomic DNA was isolated from 100 mg of approximately 3-week-old seedlings using a DNeasy Plant kit (Qiagen, http://www.qiagen.com/) according to the manufacturer's recommendations. One hundred nanograms of DNA was digested with 10 U of *Nla*III or *Dde*I, respectively, and PCRs were performed as described in Zheng *et al*. (2013). One representative experiment is shown from three biological replicates. The following primers were used: *AT5G58510* JR529 AGAGATCCGCTTCGGGAAAG and JR530 AGAAACCATTGATAGAGATGGTCTTAG, *IGN34* JR883 ATTGCCCGACGACTCCAAAT and JR884 AACTTTTGTAAGTCATGGTGTGTGTT, *IGN35* JR885 GGGCCGGGCTTAGAGGATAG and JR886 CACATCTTCTACACGTGTCTTTAGGC, *Dde*I control JR535 TCCAAGATTGAGGCCAAATTA and JR536 AAAAGGAGTGGCCAAGTTGGAA, *IGN36* JR887 GATTTTGATATTGTTACAGCATTGTT and JR888 TCCATATTCAGTACTTTTTAACCTACC, *Nla*III control JR889 ACCGTTTGTTTATGTAGGGCGAAA and JR890 AAGATAACAGAAAAGACGATGATGACG.

## Supporting Information

**Table S1.** Changes in CHH DNA methylation AGO4-bound genomic regions.
