# Supplementary material for: RNA-directed DNA methylation requires stepwise binding of silencing factors to long non-coding RNA
Source: Plant J. 2014 Jun 23;79(2):181–91. doi: 10.1111/tpj.12563 (PMC4321213; doi:10.1111/tpj.12563)
Supplement: Supplementary file 2 [file tpj0079-0181-sd2.docx]

**Supporting information legend:**

Table S1: Changes in CHH DNA methylation AGO4-bound genomic regions.
